# Green preparation of graphene oxide nanosheets as adsorbent

**DOI:** 10.1038/s41598-023-36595-2

**Published:** 2023-06-08

**Authors:** Kesheng Cao, Zhengshan Tian, Xunyou Zhang, Yabo Wang, Qiuxiang Zhu

**Affiliations:** 1 School of Chemistry and Environmental Engineering, Henan Key Laboratory of Germplasm Innovation and Utilization of Eco-Economic Woody Plant, Pingdingshan University, Weilai Road, Pingdingshan, 467000 China; 2 College of Mechanical and Electrical Engineering, Chizhou University, Chizhou, 247000 China; 3 College of Information and Electronic Engineering, Hunan City University, Yingbin East Road, Yiyang, 413000 China

**Keywords:** Chemistry, Materials science

## Abstract

As a basic building block of graphene-based materials, graphene oxide (GO) plays an important role in scientific research and industrial applications. At present, numerous methods have been employed to synthesize GO, there are still some issues that need to be solved, thus it is of importance to develop a green, safe and low-cost GO preparation method. Herein, a green, safe and fast method was designed to prepare GO, namely, graphite powder was firstly oxidized in a dilute sulfuric acid solution (H_2_SO_4_, 6 mol/L) with hydrogen peroxide (H_2_O_2_, 30 wt%) as oxidant, and then exfoliated to GO by ultrasonic treatment in water. In this process, H_2_O_2_ was the only oxidant, and no other oxidants were used, thus the explosive nature of GO preparation reaction in the conventional methods could be completely eliminated. This method has other advantages such as green, fast, low-cost and no Mn-based residues. The experimental results confirm that obtained GO with oxygen-containing groups has better adsorption property compared to the graphite powder. As *adsorbent*, GO can remove methylene blue (50 mg/L) and Cd^2+^ (56.2 mg/L) from water with removal capacity of 23.8 mg/g and 24.7 mg/g, respectively. It provides a green, fast and low-cost method to prepare GO for some applications such as adsorbent.

## Introduction

Graphene was firstly prepared by the mechanical exfoliation of highly oriented pyrolytic graphite in 2004^1^. Up to now, graphene is widely considered as one of the most important novel 2D nanomaterials^2–4^. As a derivative of graphene^5,6^, GO has its unique property due to several oxygen-containing groups covalently bonded to its basal planes and edges^7,8^. More importantly, GO acts as a basic building block, various graphene-based materials can be synthesized through the interactions between GO and other materials^9–19^. Therefore, a green, safe, fast and low-cost preparation of GO is very critical, and lots of attentions have been attracted to this issue.

On the basis of the oxidation-exfoliation process, wet chemical route^20^ is generally recognized as large-scale preparation method. Since the preparation of GO was firstly reported in 1859 based on the Brodie’s method^21^, and a mixture of potassium chlorate (KClO_3_) in fuming nitric acid (HNO_3_) was used to oxidize graphite. Nearly forty years later, Staudenmaier optimally oxidized graphite by slowly adding KClO_3_ into a mixture of fuming HNO_3_ and concentrated H_2_SO_4_^22^. In 1958, graphite was usually oxidized by the Hummers’ method^23^ with a mixture of KMnO_4_ and NaNO_3_ in concentrated H_2_SO_4_. In 2010, the Hummers’ method was further optimized and called as Tour’s method by replacing NaNO_3_ and adding proportionate *phosphoric acid* (H_3_PO_4_) as the stabilizer for the mixed system^24^. Moreover, Jin et al.^25^ used concentrated H_2_SO_4_ as intercalated molecules in the oxidation of graphite to GO and catalyst to the dehydration exfoliation of oxygenic and hydric groups from GO based on the Hummers’ method.

Although researchers have made great efforts to improve the Hummers’ method, there are some *practical issues* to be resolved, such as long reaction time, security risks, difficult quality control and a large amount of waste acid liquid^26^. Thus, numerous alternative methods have been explored to synthesize GO. For example, Gao et al.^27^ reported an iron-based method with potassium ferrate Fe (VI) (K_2_FeO_4_) as oxidant, in this process, the impurities of Mn-based metals were effectively avoided, and concentrated H_2_SO_4_ was effectively recycled. Yu et al.^28^ reported a facile and green method to synthesize GO with K_2_FeO_4_ and H_2_O_2_ as oxidants in water with pH of 3 at 50 °C. As combined catalyst, the mixture of Fe(VI) solution and H_2_O_2_ is a substitute of KMnO_4_ and strongly corrosive acids. However, these iron-based methods have some issues such as the pollution of Fe-based metals and complex post-processing.

With the environmental-friendliness and low cost, the electrochemical processes have been widely developed to prepare GO^29,30^. For example, Pei et al.^31^ used concentrated H_2_SO_4_ (98 wt%) as intercalation agent of graphite with a direct current of 1.6 V, then employed dilute H_2_SO_4_ (50 wt%) as acidic medium to oxidize the intercalated graphite with a direct current of 5 V, and finally hired ultrasonic to exfoliate the intercalated oxidized graphite. In this process, H_2_SO_4_ was primarily used as a control agent to tune the oxygen evolution reaction of water for the oxidation of graphitic lattice, thus H_2_SO_4_ was recycled, and no other oxidant was needed. Unfortunately, the accompanied water/solvent electrolysis process aggravates the expansion and delamination of graphitic materials, which lead to ineffective current supply or broken circuit^29,30^. Moreover, the structure and property of obtained products are very different from that of GO prepared by the improved Hummers’ methods^5–10^, maybe due to the low oxidation and exfoliation degree^32–35^.

At the same time, great efforts have been paid to uncover the formation mechanism of GO^36^. For example, Lee et al.^37^ exfoliated and dispersed 2D materials in pure water due to the presence of surface charges as a result of edge functionalization or intrinsic polarity, which can induce the oxygen attacking. Li et al.^38^ exfoliated large graphite crystals to small graphene flakes due to the sonication leading to the rupture and kink band striations on the flake surfaces, subsequent oxidative attack and intercalation of solvent. More recently, Zhu et al. *exquisitely* prepared GO sheets by the microfluidic oxidation of graphite with KMnO_4_ in concentrated H_2_SO_4_ in a sealed screw-top bottle due to the enhanced mass transfer and extremely quick energy exchange^39^.

On the basis of the development of GO preparation methods and the exploration of GO formation mechanism above mentioned, we are trying to find a green, safe and low-cost way to prepare GO. In this paper, a green, safe and low-cost method was designed to synthesize GO by oxidizing graphite powder in a dilute H_2_SO_4_ solution with H_2_O_2_ as oxidant, and subsequent ultrasonic treatment in water. The experimental results prove that GO can be prepared by this method. Interestingly, the obtained GO with oxygen-containing groups can be used as adsorbent to remove methylene blue and Cd^2+^ from water.

## Experimental section

### Materials

Graphite powder (99.8 wt% purity, 200 mesh), H_2_SO_4_ (18 mol/L) and H_2_O_2_ (30 wt% in water) were purchased from National Pharmaceutical Reagent Company. All chemicals were used without further purification. Deionized water (a resistance of 18 MΩ) made from a Milli-Q solvent system was used through all the experiments.

### Preparation of GO

As a typical process, the graphite powder was firstly oxidized in a mixture of H_2_SO_4_ and H_2_O_2_, and then exfoliated to GO in water.

Firstly, 2.0 g of graphite powder was added into a beaker with 50 mL of dilute H_2_SO_4_ solution (6 mol/L), and 20 mL of H_2_O_2_ (30 wt%) was slowly added into the mixed dispersion under electromagnetic stirring condition. Then, the mixed dispersion was heated to 40 ℃ through a temperature control water bath for 3 h. As control experiments, another beaker was added with 2.0 g of graphite powder and 50 mL of H_2_SO_4_ solution without adding H_2_O_2_, following the same experimental operation as that of the first beaker.

Secondly, after cooling naturally to room temperature, the resulting dispersion was filtered and cleaned with deionized water several times to pH of 7. The dilute H_2_SO_4_ solution was collected and stored for reuse.

*Thirdly*, a portion of sample was treated with ultrasound in deionized water for 30 min, after filtering, the obtained GO was dried in vacuum at 60 °C for characterizations.

In this process, only H_2_O_2_ was oxidizer, and no other oxidant was used. Dilute H_2_SO_4_ solution was primarily used as an acidic medium^28,31^ and control agent to tune the oxygen radical reaction, thus H_2_SO_4_ was recycled. The preparation process takes less than 4 h, including 3 h of water bath at 40 °C for oxidation reaction and 30 min of ultrasonic stripping at room temperature.

### Characterizations

The microstructures of graphite powder, intermediate and obtained GO were detected by the scanning electron microscopy (SEM, Hitachi S-4800), transmission electron microscopy (TEM, JEM-2100), and atomic force microscopy (AFM, Nanoscope V, Bruker Instruments). The X-ray diffractometer (XRD, Bruker D8 diffractometer), Fourier transform infrared spectroscopy (FTIR, Nicolet5700), Raman spectrometer (LabRAM HR800) and X-ray photoelectron spectroscopy (XPS, K-alpha1063) were used to analyze the structural composition of GO.

## Results and discussion

### Structure analysis

**Figure 1:**
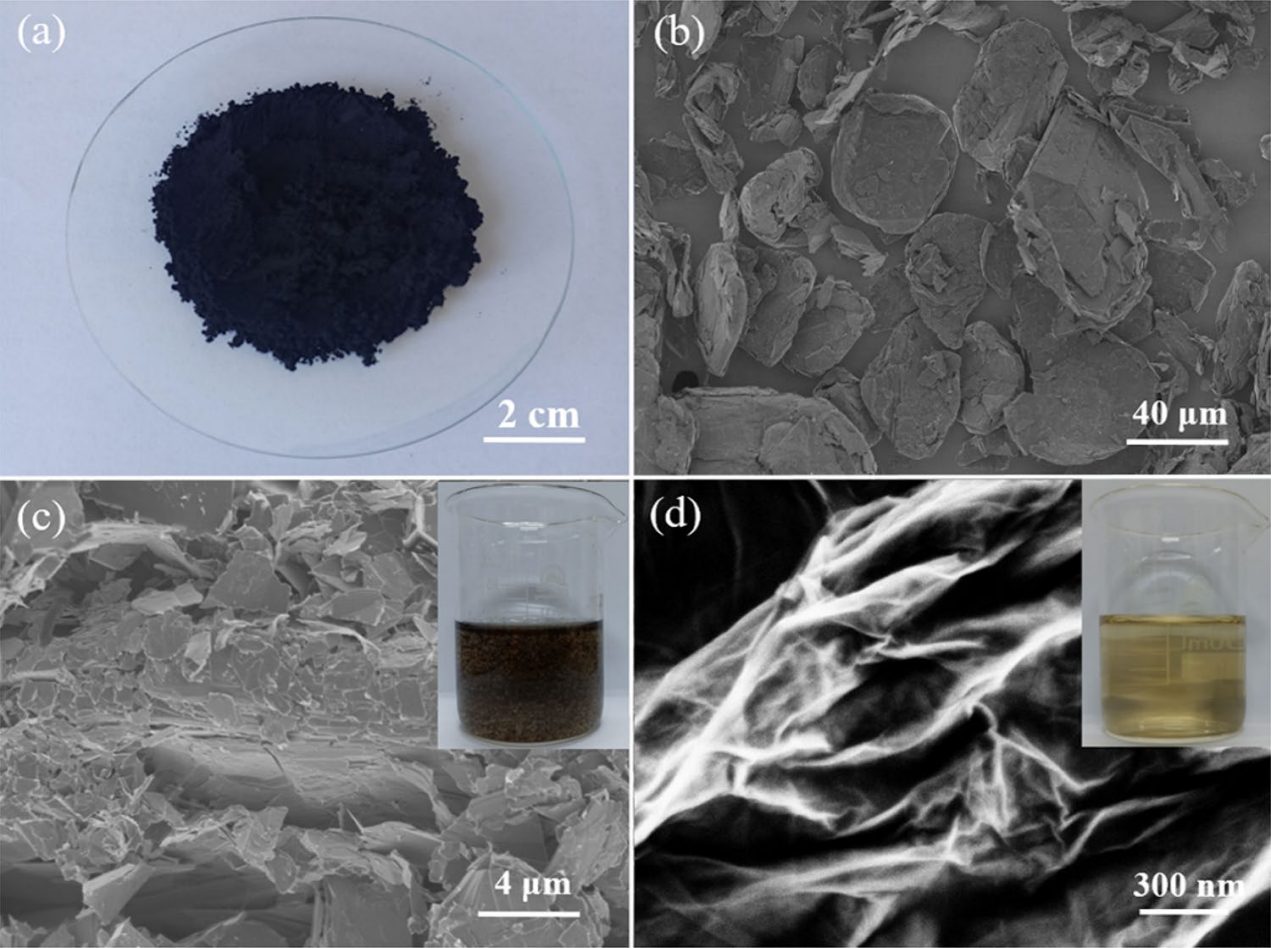
(**a**) Optical photograph of graphite powder. SEM images (**b**) of graphite powder, (**c**) intermediate and (**d**) GO. The inset of (**c**) is a dispersion of intermediate, and the inset of (**d**) is a solution of GO (0.5 mg/mL).

The microstructures of graphite powder, oxidized graphite (marked as intermediate) and GO are shown in Fig. 1. The graphite powder has irregular and tightly stacked structure (Fig. 1b), while the structure of intermediate is loose (Fig. 1c), very different from that of graphite powder, and it can be easily scattered in the deionized water (the inset of Fig. 1c). After ultrasound treatment in deionized water, the intermediate can be exfoliated to GO (Fig. 1d), and the color of GO solution is light yellow (the inset of Fig. 1d).

**Figure 2:**
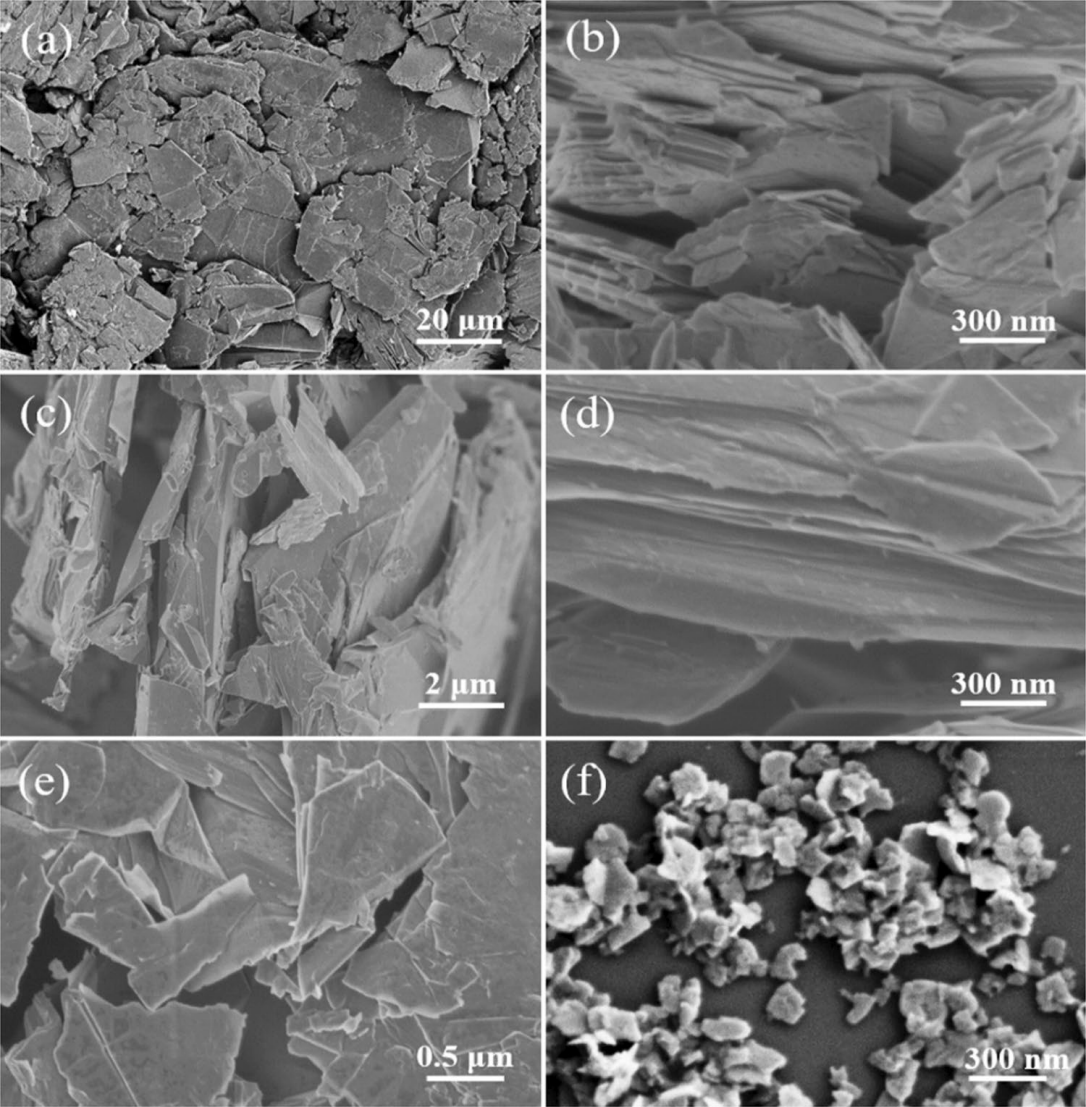
SEM images of graphite powder (**a**, **c**) and the intermediate (**b**, **d**) with different magnifications. SEM images of (**e**) large sheets derived from graphite powder and (**f**) GO nanosheets derived from the intermediate.

Figure 2a further reveals that graphite powder has irregular and stacked structure with sizes in a range of dozens of microns. In contrast, the intermediate has a loose structure with smaller sizes, as shown in Fig. 2b. Moreover, the cross-section SEM images of graphite powder and intermediate show their difference more clearly. Compared to graphite powder (Fig. 2c), the intermediate (Fig. 2d) has wider layer spacing. After ultrasonic process, the graphite powder can be separated into large sheets with a size of several microns (Fig. 2e), while the intermediate can be stripped into GO nanosheets (Fig. 2f).

**Figure 3:**
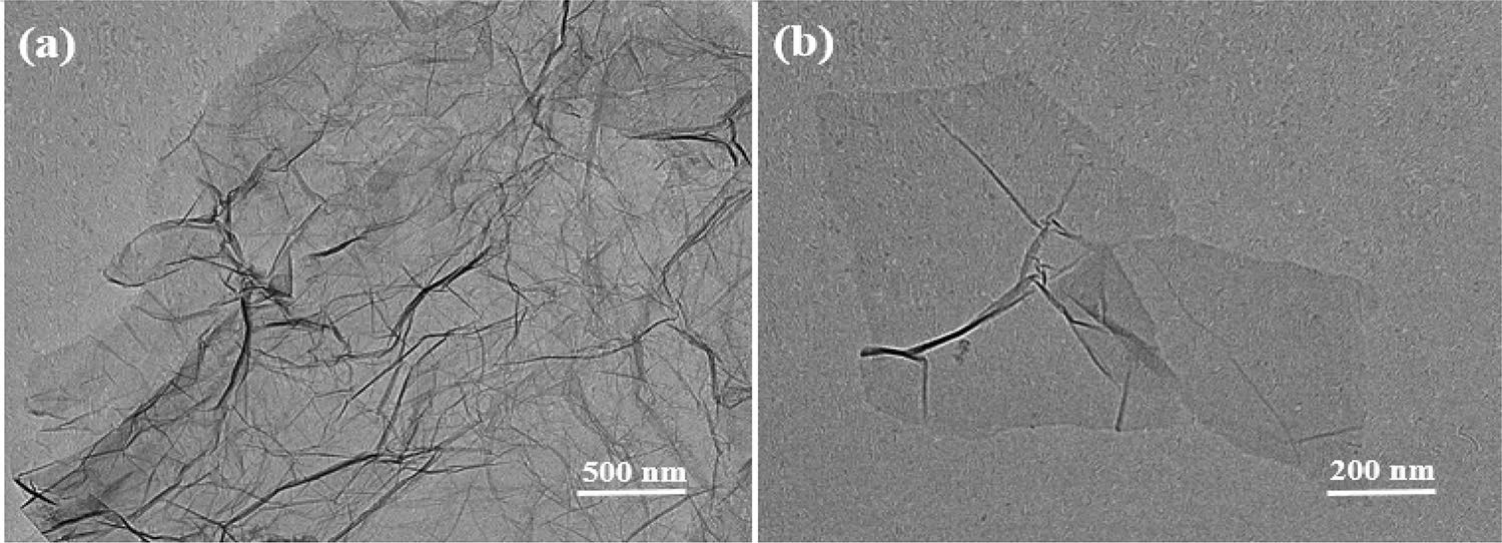
TEM images of (**a**, **b**) GO nanosheets with different magnifications.

TEM images reveal that obtained GO with folded structure is single-layer or multilayer, as shown in Fig. 3. Thus, it can be confirmed that GO nanosheets can be prepared from graphite powder by this method.

**Figure 4:**
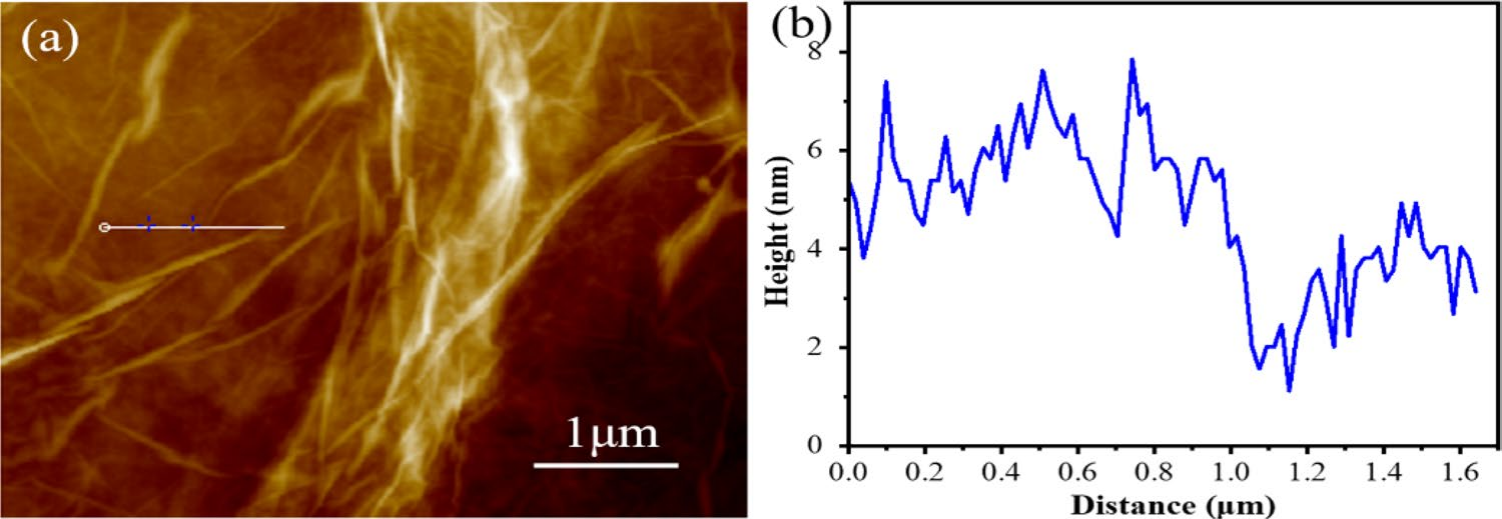
(**a**) AFM image of GO and (**b**) the corresponding height profiles of the GO surface.

After the intermediate was exfoliated to an aqueous solution of GO (0.5 mg/mL) by ultrasonic treatment, and then GO solution was dripped onto freshly cleaned Si substrate after vacuum drying for AFM characterization. Figure 4 shows that GO with a wrinkled structure has a few-layer thickness^40^.

**Figure 5:**
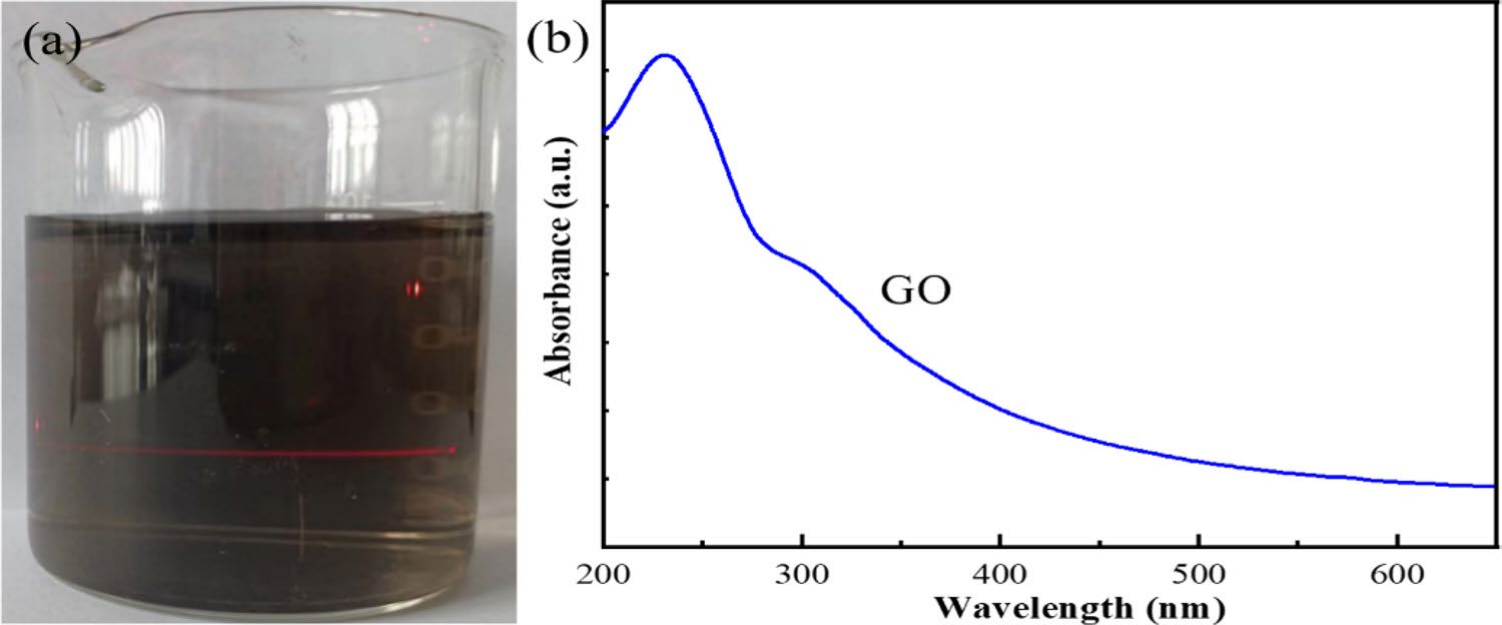
Optical photograph (**a**) and UV–Vis spectrum (**b**) of GO solution (1.0 mg/mL).

An aqueous GO solution (1.0 mg/mL) has a clear Tyndall effect (Fig. 5a). UV–Vis spectrum of this GO solution has a main peak at 231 nm and a broad shoulder at around 300 nm (Fig. 5b), suggesting that some oxygen-containing functional groups are bonded to the basal planes and edges of GO^24^.

**Figure 6:**
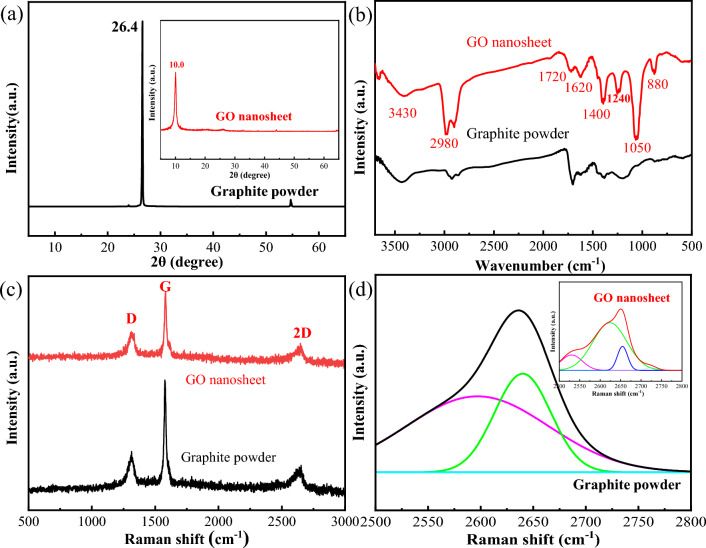
(**a**) XRD patterns, (**b**) FTIR patterns, (**c**) Raman spectra of graphite powder and GO nanosheets, and (**d**) 2D Raman peak fittings of graphite powder. The inset of (**a**) is XRD pattern of GO nanosheets, and the inset of (**d**) is 2D Raman peak fittings of GO nanosheets.

XRD, FTIR and Raman spectra of graphite powder and obtained GO nanosheets were analyzed to reveal their structural difference. As shown in Fig. 6a, the XRD peak of graphite powder locates at 2θ = 26.4°, while the peak of GO nanosheets locates at 2θ = 10.0°, and their peaks are consistent with previous reports^41,42^, indicating a larger spacing between GO layers compared with graphite powder.

Based on the FTIR spectra analyses, the obtained GO nanosheets have some functional groups^41,42^ such as O–H stretching vibration (3430 cm^−1^), C=O stretching vibration (1720 cm^−1^), C=C vibration (1620 cm^−1^), C–O vibrations (1400 cm^−1^, 1050 cm^−1^) and C–OH vibration (1240 cm^−1^), while the FTIR spectra of graphite powder are different from that of obtained GO (Fig. 6b).

Raman analyses were also used to prove their structural change (Fig. 6c). It can be seen that both of them have D peak (∼ 1320 cm^−1^), G peak (∼ 1580 cm^−1^) and 2D band (∼ 2650 cm^−1^). Moreover, the intensity ratio of the D band (∼ 1320 cm^−1^) to the G band (∼ 1580 cm^−1^) (*I*_*D*_*/I*_*G*_) slightly increases from 0.38 for graphite powder to 0.45 for the obtained GO nanosheets, indicating that the crystal defects and disorder are increased in the obtained GO nanosheets^41,42^. Moreover, their 2D Raman peak fittings have clear difference, as shown in Fig. 6d.

**Figure 7:**
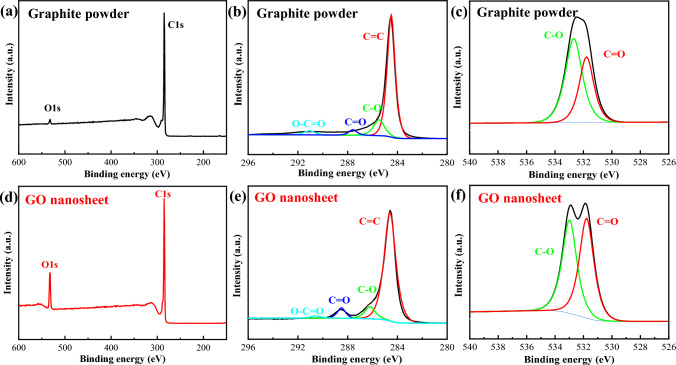
(**a**) XPS survey spectra, (**b**) high-resolution C1*s* XPS spectrum and (**c**) high-resolution O1*s* XPS spectrum of graphite powder. (**d**) XPS survey spectra, (**e**) high-resolution C1*s* XPS spectrum and (**f**) high-resolution O1*s* XPS spectrum of GO nanosheets.

The XPS spectra of graphite powder and GO nanosheets were performed to analyze their composition. As shown in Fig. 7, both XPS survey spectra have two clear peaks of carbon (C1*s*) and oxygen (O1*s*), while the intensity of the O1*s* peak in GO increases significantly, compared with graphite powder (Fig. 7a). The high-resolution C1s spectra reveal that both of them have C=C/C–C (284.7 eV), C–O (285.8 eV) and C=O (286.7 eV) groups^41,42^, respectively. Their high-resolution O1s XPS spectra have two major peaks located at 531.8 eV (C–O) and 533.0 eV (C=O), respectively. Moreover, the peak intensity of C=O for graphite powder (Fig. 7b,c) is lower than that of GO nanosheets (Fig. 7e,f), maybe due to the oxidation intercalation of H_2_O_2_.

### Mechanism analysis

To analyze the preparation process of GO by this method, some important factors should be taken into account as follows.

1. In the system of H_2_SO_4_/H_2_O_2_, only H_2_O_2_ acts as a strong green oxidizer, and a dilute H_2_SO_4_ solution (6 mol/L) is an acidic medium. In this process, some oxygen radicals (such as ·OH and ·OH_2_) can be produced from H_2_O_2_ under acidic conditions, and then the creases and defects on the graphite surface are selectively attacked by oxygen radicals^37,38^. As a result, the selective attacking endowed GO with some oxygen-containing groups, which are confirmed by the FTIR and XPS spectra of graphite powder and GO nanosheets. Dilute H_2_SO_4_ solution is primarily used as an acidic medium^28,31^ and control agent to tune the oxygen radical reaction, thus H_2_SO_4_ is recycled.
2. The oxidant content and the reaction temperature are very critical influencing factors. In this process, if the oxidant content is low, and the desired oxidation effect will not be achieved. On the other hand, if the temperature of the reaction is too high, and the acceleration of the decomposition reaction of H_2_O_2_ results in insufficient reaction time. After optimization, the mixed dispersion (including 2.0 g of graphite powder, 50 mL of 6 mol/L H_2_SO_4_ solution, and 20 mL of 30 wt% H_2_O_2_) was in a beaker and heated to 40 °C for 3 h, thus the graphite surface was attacked by enough oxygen radicals derived from the decomposition of H_2_O_2_ for enough time.
3. The GO was prepared by this method involving two main processes: the oxidation intercalation and ultrasonic delamination. The oxidation intercalation was accomplished by the selective attacking of oxygen radicals in a dilute H_2_SO_4_ solution, which causes GO surface with lots of oxygen-containing functional groups. The delamination was completed by ultrasonic treatment in deionized water, and no other intercalation agent was required in this process.

According to the above analysis, only H_2_O_2_ was used as oxidant without harmful byproducts, the dilute H_2_SO_4_ solution was used as acidic medium and recycled. The dispersion containing intermediate was not viscous, and the intermediate was easily filtered and cleaned for delamination, thus the preparation process was green, safe and low-cost.

**Figure 8:**
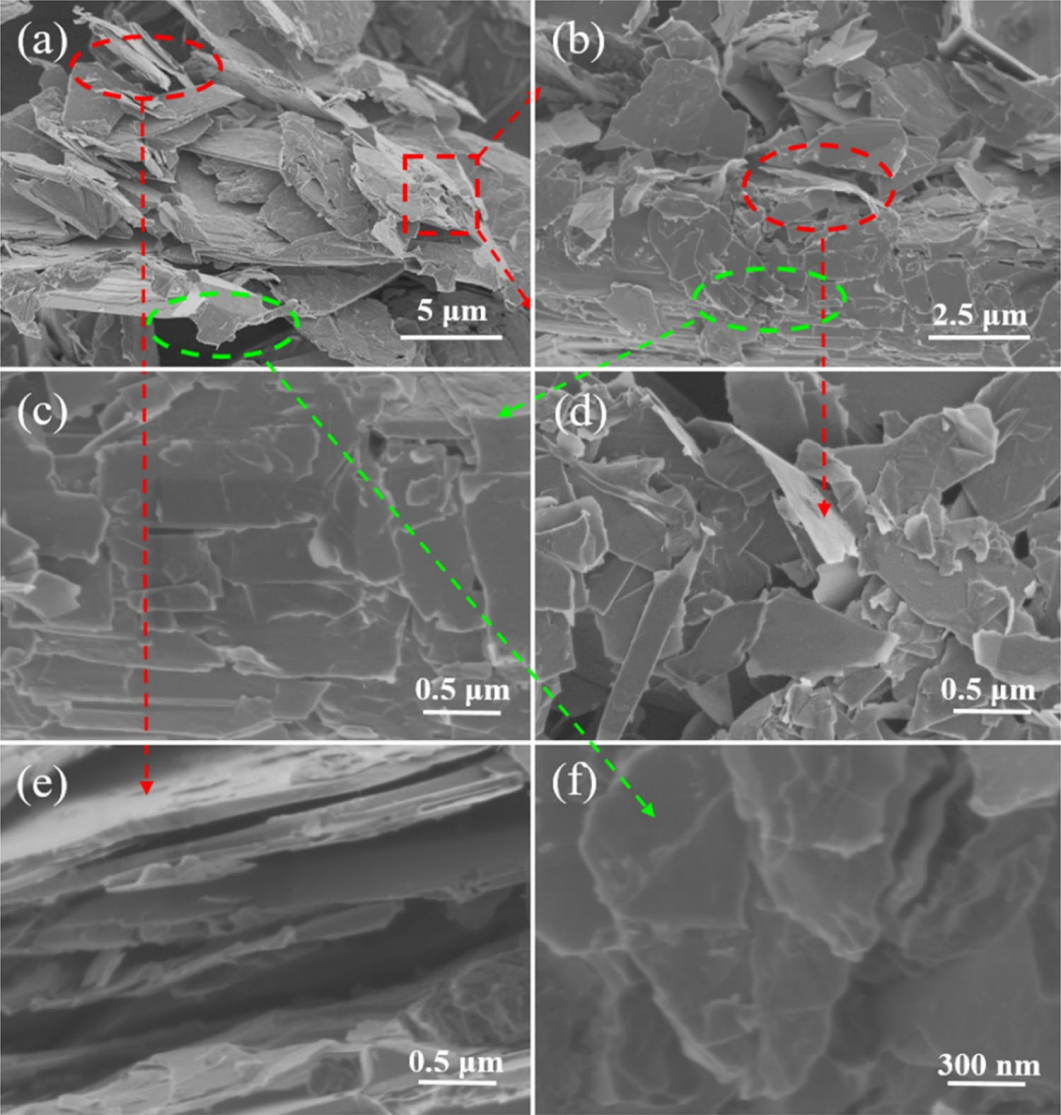
SEM images of (**a**–**d**) the intermediate with different magnifications, and (**e**, **f**) the cross-section of the intermediate.

The microstructure of the intermediate was analyzed to confirm the feasibility of this method. As shown in Fig. 8a, the intermediate shows a loose structure, compared with graphite powder (Fig. 1b). The magnified parts of its selected area clearly display some traces after selective attacking (Fig. 8b–d). Moreover, the spacing between layers was markedly widened in the intermediate (Fig. 8e), and lots of traces were left at the edges after selective attacking (Fig. 8f), thus GO nanosheets were successfully prepared from graphite powder to intermediate after the oxidation intercalation of H_2_O_2_ and ultrasonic treatment in deionized water.

### Adsorptive property

The obtained GO nanosheets with abundant functional groups can be used as adsorbent to remove methylene blue (MB) and Cd^2+^ from water. In general, MB was used as the model compound to verify the adsorption property of adsorbent. The general experimental process was described as follows: 110 mg of adsorbent was added to 50 mL of MB solution (50 mg/L), followed by stirring at 200 rpm at room temperature. At time intervals of 10 min, the adsorbent was separated by filtration and the concentration of residual MB in the filtrate was calculated by Beer’s law based on the absorption peak at 662 nm with a UV–Vis spectrophotometer (Shimadzu UV-2450)^43,44^.

On the other hand, an aqueous solution of Cd^2+^ was prepared by dissolving the corresponding lead nitrate in deionized water to arrive at a concentration of 5.0 × 10^–4^ mol/L (56.2 mg/L). Adsorption tests were carried out by using 130 mg adsorbent in 50 mL aqueous solution of Cd^2+^. Batch experiments of adsorption were carried out in conical flasks with stirring under ambient condition. At given time intervals of 10 min, 1 mL of the filtrate was obtained by filtering from the mixed solution. The concentration of Cd^2+^ in the filtrate was determined by single scan oscillopolarography (JP-303E)^45,46^.

**Figure 9:**
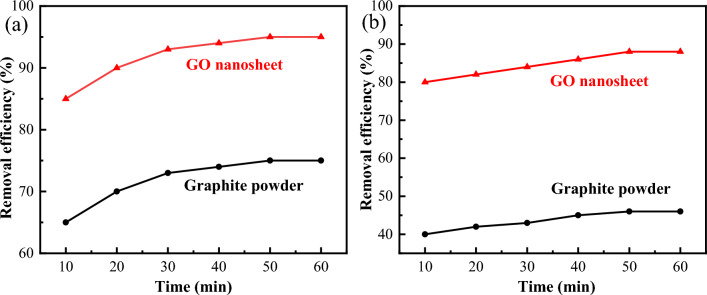
Removal efficiency of MB (**a**) and Cd^2+^ (**b**) of graphite powder and GO nanosheets.

In contrast, the graphite powder and GO nanosheets adsorbed MB (50 mg/L) at room temperature, their adsorption equilibriums reached at around 50 min (Fig. 9a), their removal efficiency are 75.0% and 95.0%, respectively. Moreover, their adsorption capacity were evaluated as 18.8 mg/g and 23.8 mg/g, respectively.

On the other hand, the graphite powder and GO nanosheets adsorbed Cd^2+^ (56.2 mg/L) from water at room temperature, their adsorption equilibriums reached at around 50 min (Fig. 9b), their removal efficiency were 46.0% and 88.0%, respectively. As well as their adsorption capacity were evaluated as 12.9 mg/g and 24.7 mg/g, respectively.

From the above comparison of adsorption properties, the graphite powder and GO nanosheets can adsorb MB and Cd^2+^ from water, especially the adsorption capacity of GO nanosheets is higher than that of graphite powder and some results reported^47–51^. The adsorption results were also compared with the literature^47–55^. The adsorption characteristics of MB and Cd^2+^ onto GO or GO composites were shown in Table 1. In our experiment, GO was directly used as adsorbent, and its adsorption property should be improved. In particular, GO can be hybridized to fabricate composite materials with better adsorption property. For example, the GO/chitosan sponge with chitosan sponge content of 9% has adsorption capacity of 275.5 mg/g for MB^56^.

**Table 1:** Adsorbents for removal of MB and Cd(II).

Adsorbents	Pollutants [MB or Cd(II)]	Adsorption capacity (mg/g)	Ref.
N-RGO	MB	94.4	^44^
Biomass activated carbon	MB	24.0	^52^
GO	MB	17.3	^47^
GO/polyaniline	MB	6.70	^48^
TiO_2_/GO composites	MB	5.01	^49^
GO	MB	23.8	This work
GO/CNTs membranes	Cd(II)	48.0	^53^
RGO	Cd(II)	24.47	^54^
GO	Cd(II)	23.90	^55^
GONH_2_	Cd(II)	10.04	^50^
G-COOH	Cd(II)	3.37	^51^
GO	Cd(II)	24.7	This work

Due to abundant functional groups and structure defects on its base planes and edges, GO has unique structure and property (such as novel physicochemical property, large specific surface area, and highly active surface), which plays an important role in the removal of organic and inorganic pollutants from water^57^.

In the case of MB adsorption, negatively charged GO interacts with positive dye of MB, thus the electrostatic attraction and hydrophobic π-π interactions are responsible for this adsorption process^58,59^. Moreover, the adsorption kinetics and thermodynamics of MB onto GO was demonstrated as a mixed physisorption-chemisorption process^60^.

To remove heavy metal ions from water, GO has various interactions such as coordination, chelation, electrostatic interaction, π–π interaction, acid base interaction with various metal/metal ions, due to its unique chemical structure containing various hydrophilic containing-oxygen groups and tiny *sp*^2^ carbon domains surrounded by *sp*^3^ domains^61–63^. Bsaed on the literature reported^64–66^, the adsorption process of Cd^2+^ onto GO was consistent with the pseudo second-order equation and the Langmuir isotherm model. However, the actual adsorption process has various influencing factors^67^, such as the electronegativity and standard reduction potential of heavy metal ions.

On the other hand, the experimental conditions play an important role in the specific adsorption process. In the cases of MB and Cd^2+^, some importan influence factors (such as adsorbent dose, solution pH, contact time and temperature) should be taken into account as follows. As shown in Fig. 9, their adsorption equilibriums reach at around 50 min, and the adsorption contact time is considered to be extended to one hour. In order to save operating costs, the adsorption temperature is kept at room temperature.

At the same time, the dosage of adsorbent is gradually increased from 50 to 70, 90, 110, 130, 150 and 170 mg in 50 mL of MB solution (50 mg/L) or Cd^2+^ solution (56.2 mg/L), and the pH of solution is gradually adjusted from 1 to 12.

**Figure 10:**
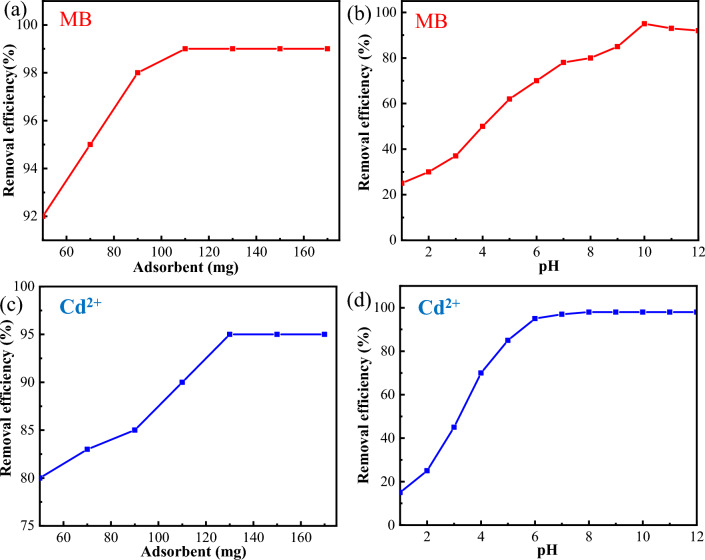
(**a**, **b**) Effects of adsorbent concentration and pH of solution on MB adsorbed onto GO, and (**d**, **f**) Effects of adsorbent concentration and pH of solution on Cd^2+^adsorbed onto GO.

Figure 10a shows that adsorption removal of MB reaches equilibrium as the dosage of adsorbent is 110 mg in 50 mL solution, while Fig. 10b reveals that adsorption removal of MB reaches peak at pH of 10. On the other hand, Fig. 10c shows that adsorption removal of Cd^2+^ reaches equilibrium as the dosage of adsorbent is 130 mg in 50 mL solution, while Fig. 10d reveals that adsorption removal of Cd^2+^ reaches peak at pH of 7.

## Conclusion

In conclusion, we develop a green, safe, fast and low-cost method to prepare GO nanosheets by oxidizing graphite powder in a dilute H_2_SO_4_ solution with H_2_O_2_ as oxidant and subsequent ultrasonic stripping in deionized water. The formation mechanism can be attributed to strong oxygen radicals attacking to creases and defects on the surface of large graphite sheets, which cause intermediate with oxygen-containing functional groups to be easily exfoliated into nanosheets by ultrasonic treatment. This work provides a green, safe and low-cost method to prepare GO nanosheets for functional applications such as adsorbent.

## Data Availability

All data generated or analyzed during this study are included in this article.
